# 561 Three-Year Follow Up Treatment For Major Full-Thickness Burns Using Dermal Biodegradable Substitute and Autologous-Skin-Cell-Suspension

**DOI:** 10.1093/jbcr/irad045.157

**Published:** 2023-05-15

**Authors:** Stephanie Chang, Aldin Malkoc, David Wong

**Affiliations:** Arrowhead Regional Medical Center, Colton, California; Arrowhead Regional Medical Center, Colton, California; Arrowhead Regional Medical Center, Colton, California; Arrowhead Regional Medical Center, Colton, California; Arrowhead Regional Medical Center, Colton, California; Arrowhead Regional Medical Center, Colton, California; Arrowhead Regional Medical Center, Colton, California; Arrowhead Regional Medical Center, Colton, California; Arrowhead Regional Medical Center, Colton, California

## Abstract

**Introduction:**

Novel techniques and technologies have shown increased survival in large TBSA burns, however, challenges with meaningful quality of life and functional outcome pose a concern. Dermal substitute and skin cell suspensions provide an area of ongoing investigation to reduce morbidity from scars, loss of functionality from contractures, and need for further procedures. This study is a follow up on the use of a dermal substitute along with autologous skin cell suspension (ASCS) with long term physical and mental outcomes as assessed with the Burn Specific Health Scale in two survivors of greater than 90% TBSA full-thickness burns.

**Methods:**

We report on two 18-year-old patients treated with the use of allograft, dermal substitute, and ASCS with split-thickness skin grafts (STSG) to achieve coverage of >90% burns. Patient 1 initially had full-thickness “leathery” burns, sparing only the plantar and dorsal aspect of the feet and patient 2 had fourth-degree burns in most areas with only the groin and feet spared. The patients’ clinic visits were reviewed and both patients agreed to participate in the Brief Burn Specific Health Scale (BSHS-B) questionnaire three-years post discharge. The survey responses of each patient were separated into their respective BSHS-B health domains and a raw Health-related quality of life (HRQOL) score was calculated for each domain.

**Results:**

Patient survey responses were categorized into their respective domains: interpersonal relationships, simple abilities, affect, body image, sexuality, hand function, treatment regimens, heat sensitivity, and work. We observed in both patients that the hand function and heat sensitivity domains of the patient’s BSHS-B were the lowest scoring in their survey responses, while the interpersonal relationships and simple abilities were the highest scoring. 15 months post discharge the patients’ bilateral upper extremities had 130° of flexion, 80° of supination, and 80° of pronation with no instability of the elbow. Both patients were continuing to work with physical therapy and were able to ambulate in their own home.

**Conclusions:**

Appropriate psychosocial management in addition to aggressive targeted surgical management and regular post-discharge clinical follow-ups were noted to have contributed to the improved post-operative outcomes in this patient with extensive burns. The findings reported in this follow-up case series demonstrate the promise of utilizing a dermal substitute and ASCS with STSG in acute surgical management of patients with extensive burns. This follow-up study demonstrates the importance of aggressive physical rehabilitation and psychosocial management in these patients’ post-operative course of recovery.

**Applicability of Research to Practice:**

The findings reported in this follow-up case series suggest the importance of dermal substitute and ASCS is the enhanced positive psychosocial domains and adequate cosmetic appearance along with enhanced physical healing.

